# Apical Localization of Inositol 1,4,5-Trisphosphate Receptors Is Independent of Extended Synaptotagmins in Hepatocytes

**DOI:** 10.1371/journal.pone.0114043

**Published:** 2014-12-01

**Authors:** Maria Jimena Amaya, André G. Oliveira, Lena K. Schroeder, Edward S. Allgeyer, Joerg Bewersdorf, Michael H. Nathanson

**Affiliations:** 1 Section of Digestive Diseases, Department of Internal Medicine, Yale University, New Haven, Connecticut, United States of America; 2 Department of Cell Biology, Yale University, New Haven, Connecticut, United States of America; 3 Department of Biomedical Engineering, Yale University, New Haven, Connecticut, United States of America; 4 Kavli Institute for Neuroscience, Yale University, New Haven, Connecticut, United States of America; Cornell University, United States of America

## Abstract

Extended synaptotagmins (E-Syts) are a recently identified family of proteins that tether the endoplasmic reticulum (ER) to the plasma membrane (PM) in part by conferring regulation of cytosolic calcium (Ca^2+^) at these contact sites (Cell, 2013). However, the mechanism by which E-Syts link this tethering to Ca^2+^ signaling is unknown. Ca^2+^ waves in polarized epithelia are initiated by inositol 1,4,5-trisphosphate receptors (InsP3Rs), and these waves begin in the apical region because InsP3Rs are targeted to the ER adjacent to the apical membrane. In this study we investigated whether E-Syts are responsible for this targeting. Primary rat hepatocytes were used as a model system, because a single InsP3R isoform (InsP3R-II) is tethered to the peri-apical ER in these cells. Additionally, it has been established in hepatocytes that the apical localization of InsP3Rs is responsible for Ca^2+^ waves and secretion and is disrupted in disease states in which secretion is impaired. We found that rat hepatocytes express two of the three identified E-Syts (E-Syt1 and E-Syt2). Individual or simultaneous siRNA knockdown of these proteins did not alter InsP3R-II expression levels, apical localization or average InsP3R-II cluster size. Moreover, apical secretion of the organic anion 5-chloromethylfluorescein diacetate (CMFDA) was not changed in cells lacking E-Syts but was reduced in cells in which cytosolic Ca^2+^ was buffered. These data provide evidence that E-Syts do not participate in the targeting of InsP3Rs to the apical region. Identifying tethers that bring InsP3Rs to the apical region remains an important question, since mis-targeting of InsP3Rs leads to impaired secretory activity.

## Introduction

One of the primary functions of intracellular Ca^2+^ signaling in polarized epithelia is the regulation of fluid and electrolyte secretion [1]–[3]. Ca^2+^ signals in these cells are organized as polarized Ca^2+^ waves that are initiated apically due to local clustering of the inositol 1,4,5-trisphosphate receptor (InsP3R) Ca^2+^ release channel [4], [5]. This apical targeting of InsP3Rs creates a “trigger zone” that allows local increases in Ca^2+^ concentration [4], [6]–[8], which are important for exocytosis [9], the insertion of key membrane transporters into the apical membrane [10], [11] and their function [12], [13], which together drive the secretory activity of these cells.

There are three isoforms of the InsP3Rs, namely I, II and III [14]–[16]. Some polarized epithelial cells, including hepatocytes and bile duct cells (or cholangiocytes), have one principal isoform tethered to the apical membrane [4], [6] while others, such as pancreatic acinar cells, have more than one [17]. In either case, loss of apical InsP3R expression, whether due to decreased InsP3R expression [18] or redistribution away from the apical region [19], leads to impaired Ca^2+^ signaling and consequently impaired secretion [10], [11], [18], [20]. Moreover InsP3R deficiency is a common feature in patients with different types of secretory diseases [18].

Despite the importance for cell function, the exact mechanism that tethers InsP3Rs to the apical membrane remains to be determined. There is evidence that the apical localization of InsP3Rs and the function of the “trigger zone” depends upon the integrity of detergent-resistant membranes or lipid rafts, suggesting that these structures act as signaling microdomains that ensure the proper targeting of these receptors [19]. However, it is not clear whether tethering proteins are necessary to target InsP3Rs to these domains of the apical membrane.

Extended Synaptotagmins (E-Syts), which are homologous to tricalbins in yeast, are recently identified and characterized ER integral membrane proteins that contain a cytosolic synaptotagmin-like mitochondrial lipid binding protein (SMP) domain (a lipid-binding module that is thought to mediate lipid exchange between the ER and the PM), followed by multiple C2 domains (Ca^2+^ and phospholipid-binding modules) [21], [22]. These tethers allow the formation of ER-PM contacts through the InsP3 precursor PI(4,5)P_2_ and the regulation of cytosolic Ca^2+^ [23], [24]. Here we investigated whether E-Syts participate in the tethering of the InsP3R to the apical membrane in hepatocytes, a model of polarized epithelial cells in which the machinery for calcium signaling and secretion has been carefully defined [4], [10], [11].

## Materials and Methods

### Animals and materials

Male Sprague-Dawley rats weighing 180–250 g (Charles River Labs, Wilmington, MA) were used for all experiments. All animal procedures were approved by the Yale Animal Care and Use Committee. TaqMan Gene expression assays containing Real Time PCR primers for rat E-Syt1, E-Syt2, E-Syt3 and GAPDH were from Life Technologies (Grand Island, NY), as well as Rhodamine phalloidin, Lipofectamine RNAiMAX and cell tracker green 5-chloromethylfluorescein diacetate (CMFDA). Rabbit E-Syt1 and E-Syt2 antibodies and small interfering RNAs (siRNAs) against E-Syt1 and E-Syt2 and scrambled negative controls were from Sigma-Aldrich (Saint Louis, MO). Mouse GAPDH antibody was from Ambion (Grand Island, NY). Rabbit InsP3R-II antibody was kindly provided by Richard Wojcikiewicz (SUNY, Syracuse, NY) [25]. Monoclonal Mrp2 antibody (M_2_ III-6) was from Alexis Biochemicals (Plymouth Meeting, PA). Hela cell lysate was from BD Biosciences (San Jose, CA). R-GECO was from Addgene (Cambridge, MA). All other chemicals were of the highest quality commercially available.

### Isolation and Collagen Sandwich Culture of Rat Hepatocytes

Cells were isolated in the Cell Isolation Core of the Yale Liver Center, as described [26], [27]. Briefly, rat livers were perfused with Hanks' A and then Hanks' B medium containing 0.05% collagenase (Roche Applied Science, Indianapolis, IN) and 0.8 units of trypsin inhibitor (Sigma-Aldrich (Saint Louis, MO) per unit of tryptic activity. Livers were minced and passed through serial nylon mesh filters, and the resultant cells were washed. Isolated hepatocytes were resuspended in complete Williams' medium E. Cells were then seeded onto collagen-I- coated coverslips and incubated at 37°C for 2 hrs before transfection with siRNAs. Cells were coated with a second layer of collagen-I 24 hrs after transfection and were used 96 hrs after plating [28]. All experimental procedures and euthanasia were approved by the Institutional Animal Care and Use Committee (IACUC).

### Real Time quantitative PCR

Total RNA was extracted from control rat hepatocytes, or from cells transfected with control or E-Syts siRNAs using RNeasy MiniKit (QIAGEN, Valencia, CA). cDNA was synthesized from 2 µg of RNA with the AffinityScript Multi Temp cDNA synthesis kit (Agilent Technologies, Santa Clara, CA). Resulting DNA was subjected to Real-Time qPCR with FastStart Universal Probe Master (Rox) (Roche, San Francisco, CA) and TaqMan Gene expression assays (Life Technologies, Grand Island, NY), according to manufacturer's instructions. Experiments were run in a 7500 Real Time PCR System (Yale Liver Center; Life Technologies, Grand Island, NY,). Quantification results were expressed in terms of the cycle threshold (Ct). All real-time qPCR reactions were run in triplicate, and the Ct values were averaged from three independent samples. Data were normalized to the reference gene GAPDH. Differences between the mean Ct values of each gene and those of the reference gene were calculated as ΔCt  =  Ct^gene^ - Ct^reference^ and represented as 2^−ΔCt^.

### Immunoblotting

Immunoblots were performed as described previously [10], [11]. Briefly, cells and whole liver pieces were lysed with mammalian protein extraction reagent (MPER) lysis buffer (Thermo Scientific, Rockford, IL) and protein concentration was determined spectrophotometrically. Thirty micrograms of total cellular protein were separated by sodium dodecyl sulfate polyacrylamide gel electrophoresis (SDS-PAGE), on a 4%–20% gel. Membranes were blocked with nonfat milk and then incubated overnight at 4°C with E-Syt1, E-Syt2, InsP3R-II or GAPDH-specific antibodies. Membranes were washed and incubated with peroxidase-conjugated secondary antibodies. Immunodetection was carried out by enhanced chemiluminescence, and blots were quantitatively analyzed using Image J (NIH, Bethesda, MD).

### Transfection of small interfering RNAs

Validated small interfering RNAs (siRNAs) for E-Syt1 and E-Syt2 and a control scrambled sequence were transfected using Lipofectamine RNAiMAX, according to the manufacturer's instructions. Cells were used 96 hrs after transfection.

### Immunofluorescence

Confocal immunofluorescence was performed as described previously [10], [11]. Briefly, hepatocytes on glass coverslips were washed and then fixed in 4% formaldehyde and permeabilized with 0.1% Triton X-100. Samples were then blocked in phosphate- buffered saline (PBS) containing 1% bovine serum albumin and 5% normal goat serum, and incubated with primary antibodies overnight at 4°C. Subsequently, samples were washed with PBS, incubated with fluorophore-conjugated secondary antibodies and Rhodamine-conjugated Phalloidin for 1 hour at room temperature, washed again with PBS, and then mounted with VectaShield (Vector Laboratories, Burlingame, CA) containing DAPI. Negative controls were incubated with secondary antibodies alone. Specimens were examined with a Zeiss LSM 710 Duo Confocal Microscope (Thornwood, NY). Cells were observed with a 40X objective and fluorescence intensity was quantified using Image J (NIH, Bethesda, MD) and Volocity Software (Perkin Elmer, Waltham, MA).

### Stimulated Emission Depletion (STED) Microscopy

Super resolution imaging was performed via gated detection, pulsed, Stimulated Emission Depletion (STED) microscopy [29] using a custom built system with spatial resolution <30 nm. The instrument is based around an 80 MHz mode-locked Ti:Sapphire laser (Chameleon Ultra II, Coherent) acting as the depletion beam tuned to 770 nm. Output pulses from the Ti:Sapphire laser (140 fs) for depletion are initially stretched by traveling through approximately 40 cm of high dispersion glass (SF6) before coupling into 23 meters of polarization maintaining photonic crystal fiber (LMA-PM-15, NKT Photonics) and finally coupled into a single mode polarization maintaining 100 meter pure silica core fiber. After exiting the optical fiber the collimated depletion beam was incident on a spatial light modulator (SLM) (X10468, Hamamatsu) conjugated to the back pupil plane of the objective lens. The SLM was used to correct for system aberrations in the depletion beam path and imprinted a 2π phase ramp on the depletion beam for formation of the so-called “doughnut”. Fluorescence excitation resulted from two pulsed diode lasers emitting at 485 and 640 nm (LDH-P-C-485B, LDH-P-C-640B, Picoquant). The 640 nm excitation diode was synchronized electronically using a custom built electronic delay with 20 picosecond step size and 19 nanoseconds total range. This same custom delay also provided detector gating with a detection window width range of 640 ps to 10 ns and a detection window that can be adjusted from 0 to 19 ns after the depletion synchronization signal is received with 20 ps step size. For STED imaging the window width was set to 6 ns and the detection window began 2 ns after the excitation pulse. The excitation and depletion beams were combined using dichroic mirrors directed onto a 16 kHz resonant scanning mirror and galvo mirror (SC-30, Electro-Optical Products Corp.) and subsequently through a quarter wave plate and into the back aperture of a 100X 1.4 NA oil immersion objective lens (UPLAPO 100XO/PSF, Olympus) resulting in diffraction limited focuses at the sample. The resonant and galvo mirrors, which were imaged into the back pupil of the objective lens, allow the excitation and depletion beam to be scanned laterally in the sample for imaging. For this application unidirectional scanning was employed. Fluorescence was collected by the same objective and de-scanned by the scanning mirrors. Dichroic mirrors were used to separate fluorescence from back-scattered excitation and depletion light and separate the ATTO647N signal from Alexa 488. After separation and passage through a band pass filter (FF01-685/40, Semrock), fluorescence was focused into 125 µm core (0.8 Airy units) multimode fiber and finally detected by a single photon counting avalanche photodiode (APD) (SPCM-ARQ-13-FC, Perkin Elmer). APD counts were collected via an FPGA based data acquisition card (PCIe-7852R, National Instruments) and processed into an image via custom microscope control software (LabVIEW, National Instrument). Due to the sinusoidal motion of the resonance mirror counts APD counts were linearized and accumulated on the FPGA card before transmission to the host PC. Imaging of InsP3R-II was performed with 50 µW of 640 nm excitation and approximately 200 mW of 770 nm depletion at the back aperture. The mean pixel dwell time was 40 ns with an image format of 512 by 512 and 20 nm pixel size. For STED, each line of the image was accumulated 800 times.

### *In vitro* secretion assay

CMFDA canalicular accumulation was monitored as described previously [11]. Briefly, coverslips containing cells were transferred to a custom-built perfusion chamber on the stage of an LSM 710 Duo confocal microscope (Zeiss, Thornwood, NY), and the cells were then perfused with 4-(2-hydroxyethyl)-1-piperazine ethanesulfonic acid-buffered (HEPES) solution containing 1µM CMFDA for 4 min. For negative controls, cells were pre-incubated with 50µM BAPTA-AM for 30 min at 37°C. Increases in canalicular organic anion secretion were expressed as CMFDA fluorescence intensity and normalized by baseline fluorescence. Cells were excited at 488 nm and observed at 505 to 550 nm. Cells were observed with a 40X objective lens.

### Detection of Ca^2+^ signals

Cells were transfected with the red fluorescent protein-tagged genetically encoded Ca^2+^ indicator for optical imaging R-GECO [30] with or without E-Syt siRNAs using Lipofectamine RNAiMAX, according to the manufacturer's instructions. Ca^2+^ imaging experiments were performed after 96 hrs of transfection. Then, coverslips containing the cells were transferred to a custom-built perfusion chamber on the stage of an LSM 710 Duo confocal microscope (Zeiss, Thornwood, NY). Cytosolic Ca^2+^ signals were monitored in R-GECO-transfected cells during stimulation with 20µM ATP (Sigma, Saint Louis, MO) using a 40X objective lens. Cells were excited at 561 nm and observed above 575 nm. Changes in fluorescence were normalized by the initial fluorescence (*F*0) and were expressed as (*F*/*F*0) x 100% [31]. Ca^2+^ signal amplitude, rise time and Ca^2+^ wave speed were calculated as previously described [32].

### Statistical analysis

Results are expressed as mean values ± standard deviation (SD) unless indicated otherwise. PRISM software (GraphPad, La Jolla, CA) was used for data analysis. Groups of data were compared using one-way analysis of variance (ANOVA), followed by Bonferroni's post-tests, and p<0.05 was taken to indicate statistical significance.

## Results

### Rat hepatocytes express extended synaptotagmins and their expression is maintained in fully polarized cells

**Figure 1:**
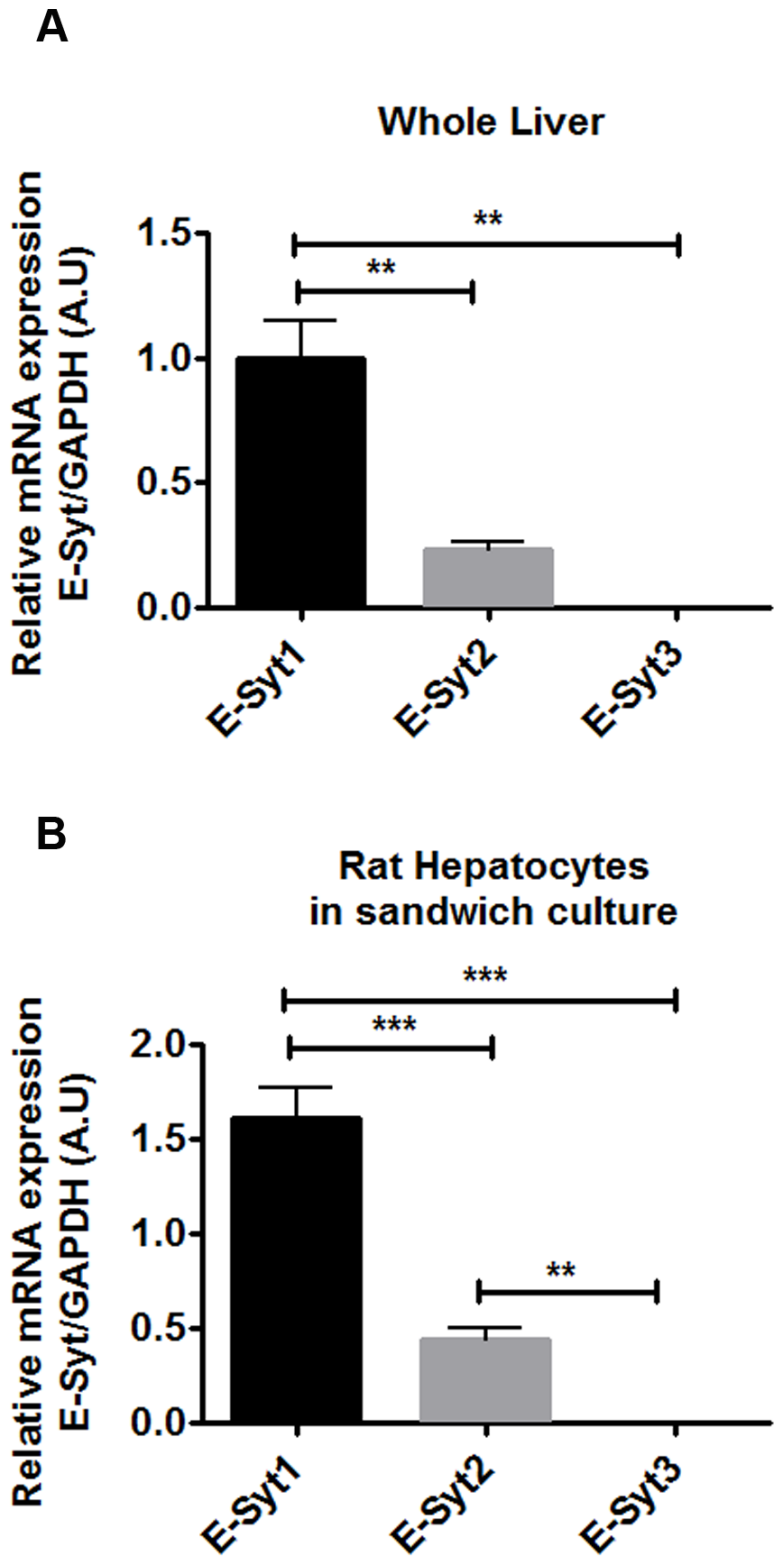
Rat hepatocytes express E-Syt1 and E-Syt2 but not E-Syt3. Relative mRNA expression of E-Syt isoforms was measured by Real Time quantitative PCR in whole liver extracts (A) and in fully polarized rat hepatocytes (after 96 hrs in sandwich culture) (B). E-Syt 1 and 2 were detected, and E-Syt3 was absent from both samples. E-Syt1 was the most abundantly expressed isoform in both samples. (**p<0.001; ***p<0.0001; n = 3 experiments). Values are mean ± SD. Data were analyzed by one-way ANOVA, followed by Bonferroni's post-tests.

**Figure 2:**
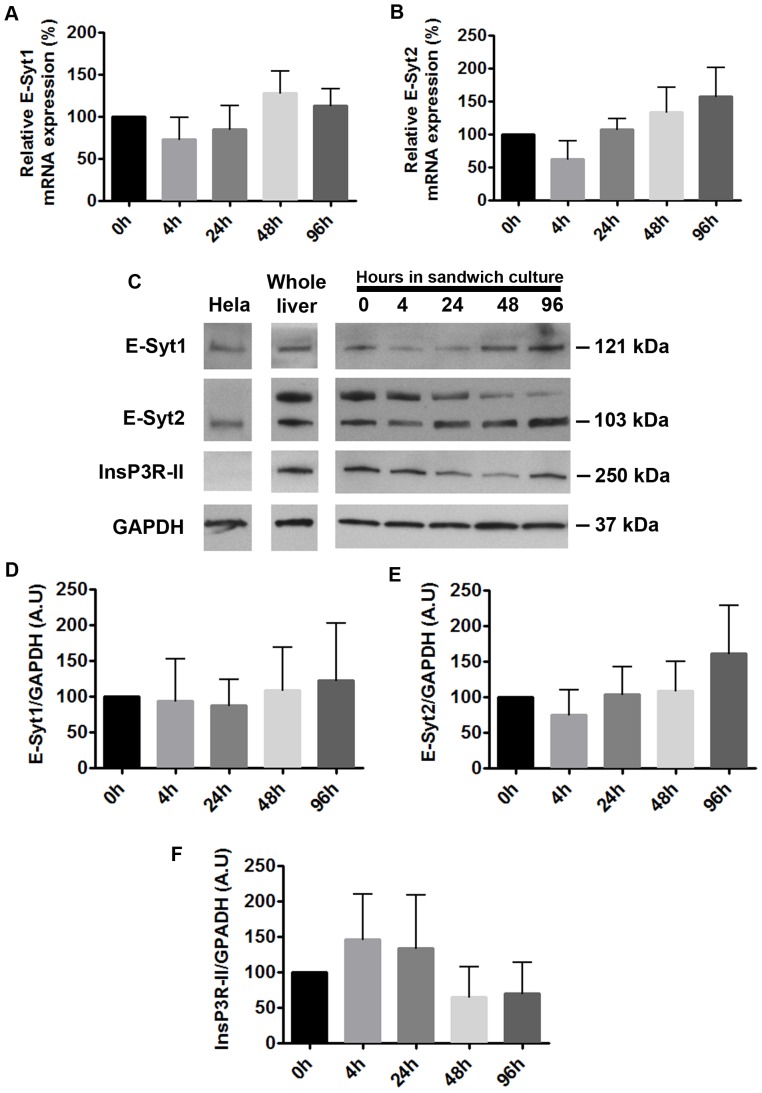
E-Syt1, E-Syt2 and InsP3RII expression is maintained during rat hepatocyte collagen sandwich culture. Relative E-Syt1(A) and E-Syt2 (B) mRNA expression was assessed by Real Time PCR in rat hepatocytes at the indicated times in collagen sandwich culture. (**C**) Representative immunoblottings of E-Syt1, E-Syt2 and InsP3R-II in rat hepatocytes at the indicated time points of collagen sandwich culture. GAPDH was used as loading control. HeLa cell lysate was used as a positive control for E-Syts and whole liver lysate was used as a positive control for InsP3R-II. Correct molecular weight is indicated by the arrows. (**D**) Densitometric analysis of E-Syt1 blots in (C). (n = 4 experiments). (**E**) Densitometric analysis of E-Syt2 blots in (C). (n = 4 experiments). (**F**) Densitomteric analysis of InsP3R-II blots in (C). (n = 4 experiments). Values are mean ± SD. Data were analyzed by one-way ANOVA, followed by Bonferroni's post-tests. Expression was compared among time points ranging from 0 to 96 hrs in collagen sandwich culture.

Three isoforms of E-Syts have been identified and characterized in mammalian cells: 1, 2 and 3 [21]–[23]. To determine which isoforms are expressed in the liver, we performed Real Time quantitaive PCR (qPCR) in whole rat liver extracts and primary rat hepatocytes in collagen sandwich culture. We used this cell system because structural and functional polarity of hepatocytes is preserved [10], [33], [34]. Additionally, polarity brings the region of the ER that is enriched in InsP3R in close proximity to the PM [19], which allows proper function of these cells [4], [10], [11]. E-Syt1 and E-Syt2 were detected, whereas E-Syt3 was absent from both whole rat liver and rat hepatocytes. Moreover, E-Syt1 was the most abundant isoform in both samples (Figure 1a-b). To determine whether E-Syt1 and E-Syt2 expression undergoes changes during rat hepatocyte collagen sandwich culture, we monitored mRNA and protein expression levels in cells after 0, 4, 24, 48 or 96 hrs of plating. We observed that E-Syt1 and E-Syt2 mRNA expression is maintained after 96 hrs in culture (Figure 2a-b), when cells reach full polarity [33], [34]. We used this time point of culture to perform functional experiments. To validate our qPCR results, immunoblots of primary rat hepatocytes at these time points after plating were performed. Consistent with our mRNA results, E-Syt1 and E-Syt2 protein expression was maintained after 96 hrs of culture relative to protein expression at time point 0 hrs. Hela cells were used as a positive control for E-Syts [23] (Figure 2c-e). Together, these data demonstrate that rat hepatocytes express E-Syt1 and E-Syt2 and that their expression is maintained in fully polarized cells in culture.

### Apical localization of InsP3Rs is independent of extended synaptotagmins

**Figure 3:**
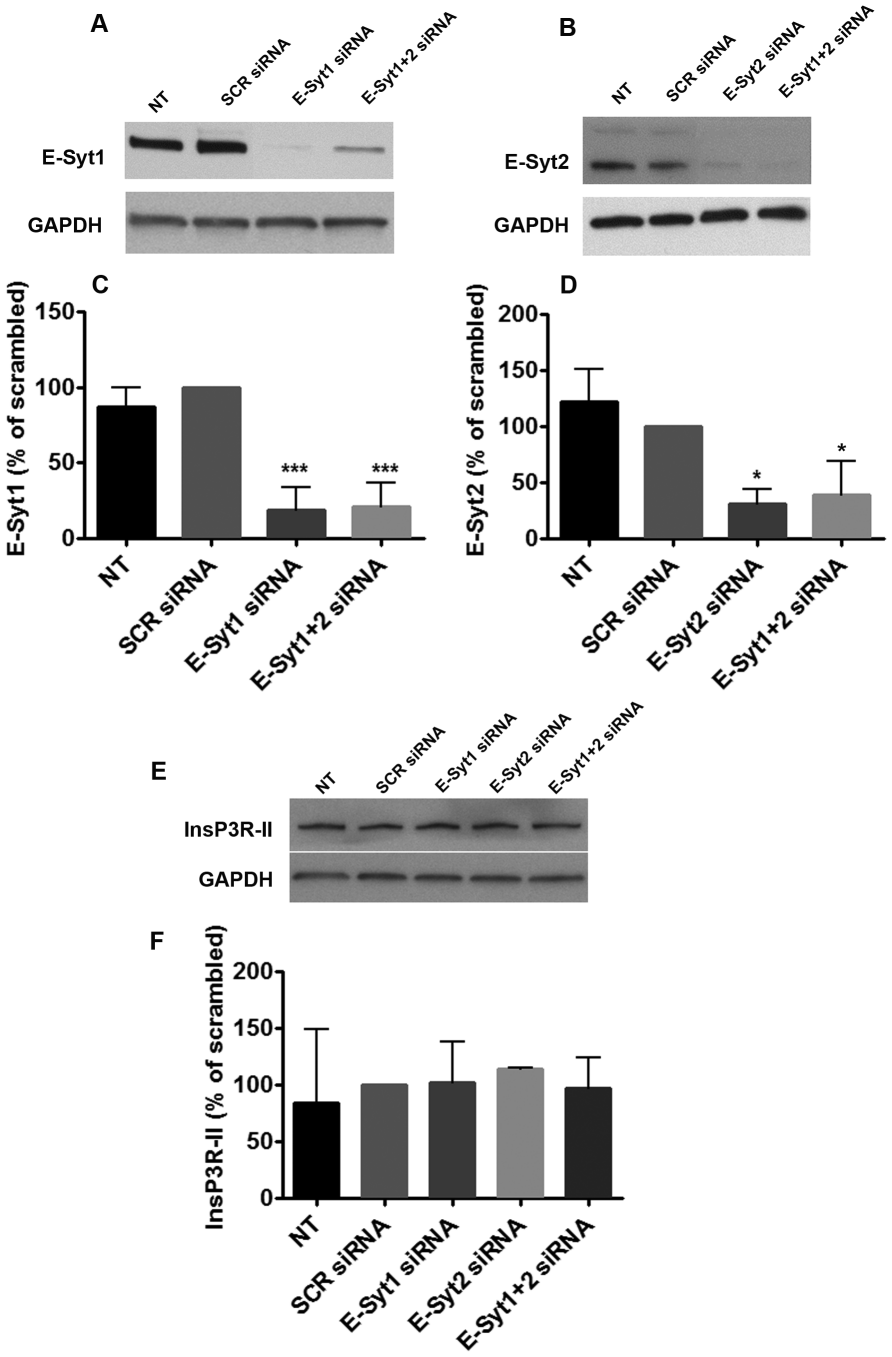
Specific siRNAs against E-Syt1 and E-Syt2 efficiently reduce E-Syt1 and E-Syt2 protein expression but do not affect InsP3R-II expression. Representative immunoblotings of E-Syt1 (A) and E-Syt2 (B) in rat hepatocytes after treatment with 25 nM of scrambled, E-Syt1 (A), E-Syt2 (B) or E-Syt1+2 siRNAs (A and B) for 96 hrs in sandwich culture. (**C**) Densitometric analysis of blots in (A). (***p<0.0001; n = 3 experiments) (**D**) Densitometric analysis of blots in (B) (*p<0.05; n = 3 experiments). (**E**) Representative immunoblots of InsP3R-II after individual or simultaneous treatment with E-Syt1 and E-Syt2 siRNAs. (**F**) Densitometric analysis of blots in (E) (n = 3 experiments). GAPDH was used as loading control. Values are mean ± SD. Data were analyzed by one-way ANOVA, followed by Bonferroni's post-tests.

**Figure 4:**
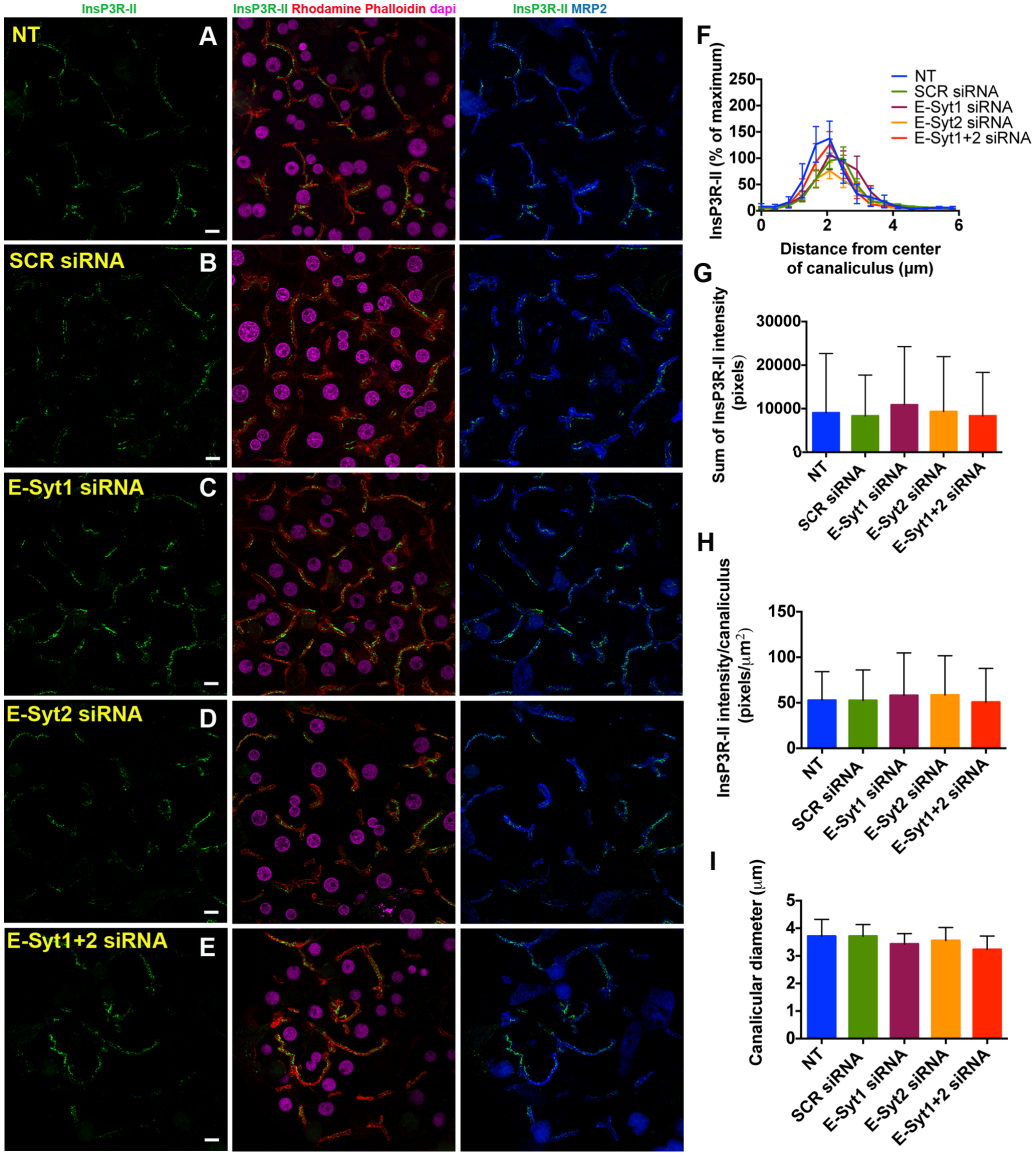
Apical localization of InsP3Rs is independent of E-Syt1 and E-Syt2. Confocal immunofluorescence images of InsP3R-II under control, non-treated (A), scrambled (B), E-Syt1 (C), E-Syt2 (D) and E-Syt1+2 (E) siRNA conditions. InsP3R-II (green) was co-labelled with Rhodamine phalloidin (red), the apical membrane marker Mrp2 (blue) and the nuclear stain DAPI (magenta). Scale bar = 10µm. (**F**) Distribution of InsP3R-II was quantified by its normalized fluorescence intensity along a 6µm line perpendicular to the canalicular membrane. (n = 3 experiments; control: n = 63 canaliculi; scrambled siRNA: n = 48 canaliculi; E-Syt1 siRNA: n = 48 canaliculi; E-Syt2 siRNA: n = 51 canaliculi; E-Syt1+2 siRNA: n = 30 canaliculi). Values are mean ± SEM. (**G**) Total InsP3R-II fluorescence intensity per canaliculus. (**H**) Relative InsP3R-II fluorescence intensity, calculated as total InsP3R-II fluorescence normalized by canalicular area. (n = 3 experiments; control: n = 234 canaliculi; scrambled siRNA: n = 134 canaliculi; E-Syt1 siRNA: n = 163 canaliculi; E-Syt2 siRNA: n = 299 canaliculi; E-Syt1+2 siRNA: n = 220 canaliculi). (**I**) Canalicular diameter is shown as an indicator of canalicular morphology. (n = 3 experiments; control: n = 63 canaliculi; scrambled siRNA: n = 48 canaliculi; E-Syt1 siRNA: n = 48 canaliculi; E-Syt2 siRNA: n = 51 canaliculi; E-Syt1+2 siRNA: n = 30 canaliculi). Values are mean ± SD. Data were analyzed by one-way ANOVA, followed by Bonferroni's post-tests.

**Figure 5:**
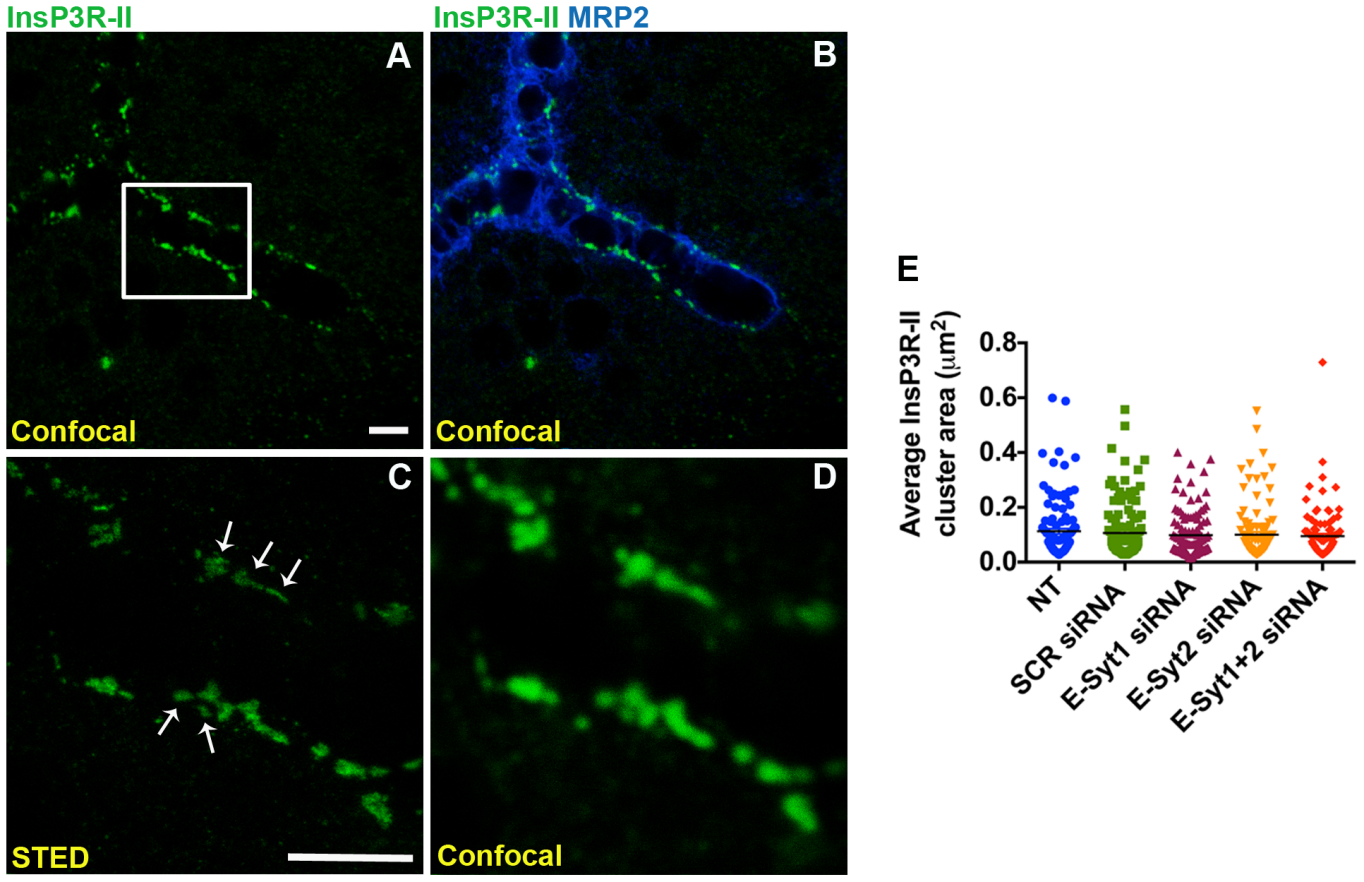
Average InsP3R-II cluster size is not altered by E-Syt knockdown. Confocal immunofluorescence (A, B, D) and STED super-resolution (C) images of a control rat hepatocyte canaliculus. InsP3R-II (green) was co-labelled with Mrp2 (blue). Scale bar = 3.5µm. Box in (A) represents the area depicted in (C) and (D). (**C**) Individual clusters (arrows) were observed in STED image, whereas they could not be resolved as well in confocal image (D). (**E**) Average InsP3R-II cluster area was determined based on STED images (control: n = 118 clusters; scrambled siRNA: n = 129 clusters; E-Syt1 siRNA: n = 118 clusters; E-Syt2 siRNA: n = 109 clusters; E-Syt1+2 siRNA: n = 103 clusters). Data were analyzed by one-way ANOVA, followed by Bonferroni's post-tests.

Ca^2+^ signaling in hepatocytes is mainly mediated by InsP3Rs, which are InsP3-gated Ca^2+^ channels localized in the ER. Two of the three isoforms of InsP3Rs are found in hepatocytes (InsP3R-I and InsP3-II). InsP3R-II is the most abundantly expressed isoform and is concentrated in the region of the ER near the apical membrane [4], [32]. Of note, InsP3R-II protein expression and subcellular localization is preserved in rat hepatocytes in collagen sandwich culture [10] (Figure 2f). In order to determine whether E-Syts mediate the targeting of InsP3R-II to the apical membrane, we used specific siRNAs that resulted in a knockdown of 82% and 69% in E-Syt1 and E-Syt2 expression, respectively compared to scrambled siRNA transfected cells (Figure 3a-d). Immunoblots of cells under individual or simultaneous E-Syt1 and E-Syt2 knockdown showed that silencing of these isoforms does not alter InsP3R-II protein expression (Figure 3e and f). We also monitored InsP3R-II apical localization by confocal immunofluorescence microscopy under these conditions, co-labeling with multidrug resistance protein 2 (Mrp2), an organic anion transporter that resides in and immediately beneath the apical membrane, and Rhodamine phalloidin, which labels f-actin and facilitates the identification of the apical membrane [11]. InsPR-II localizes in close proximity to both Mrp2 and Rhodamine phalloidin in control non-treated cells or in scrambled siRNA-treated cells [10], [11] (Figure 4a and b). InsP3R-II distribution did not change in cells treated with E-Syt1 and/or E-Syt2 siRNAs (Figure 4c-e and f). Additionally, InsP3R-II total and relative fluorescence intensity per canaliculus were not affected under E-Syt knockdown conditions (Figure 4g and h). Similarly, canalicular diameter remained unaltered in these cells (Figure 4i). In order to investigate whether E-Syt1 and/or E-Syt2 knockdown affects InsP3R-II cluster size, we used Stimulated Emission Depletion (STED) super-resolution microscopy [29]. The custom-built machine used in these studies allowed us to resolve individual InsP3R-II clusters at <30 nm resolution. Average InsP3R-II cluster area did not change in cells treated with E-Syt1 and/or E-Syt2 siRNA (Figure 5). Collectively, these results provide evidence that InsP3R-II targeting to the apical membrane of hepatocytes is independent of E-Syt 1 and E-Syt2.

### Apical organic anion secretion is not affected by E-Syt knockdown

**Figure 6:**
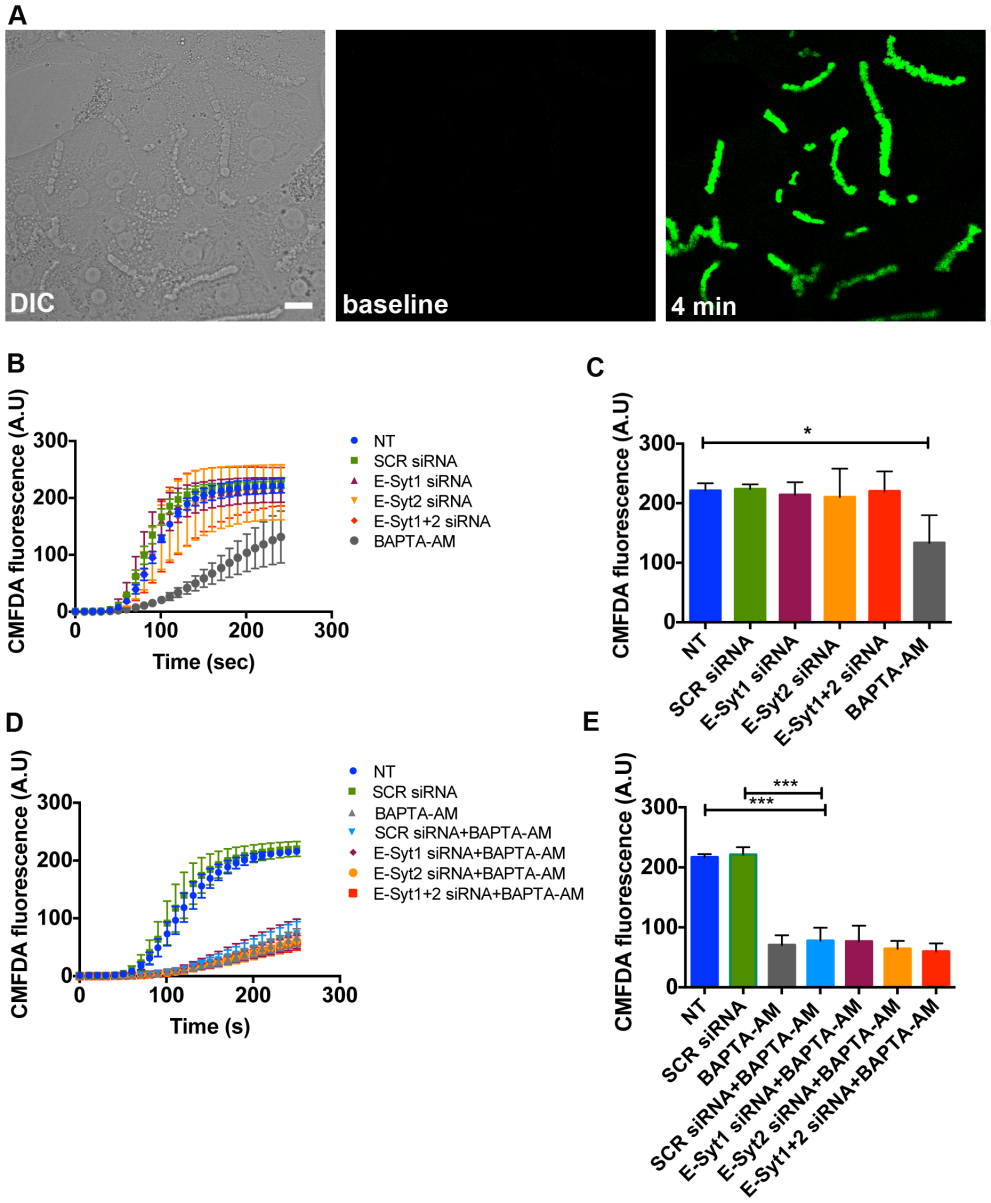
Apical organic anion secretion is not affected by E-Syt knockdown. (**A**) Representative differential interference contrast (DIC) (left), and confocal fluorescence time-lapse images at baseline (middle) and after 4 min (right) of CMFDA secretion in control non-treated rat hepatocytes in collagen sandwich culture. The cell-permeant fluorescent Mrp2 substrate was added to cells, and its secretion into the canalicular spaces (green) was monitored every second for 4 minutes by confocal microscopy. (**B**) and (**C**) Quantification of CMFDA canalicular accumulation under control (n = 55 canaliculi), scrambled (n = 49 canaliculi), E-Syt1 (n = 52 canaliculi), E-Syt2 (n = 65 canaliculi) and E-Syt1+2 (n = 52 canaliculi) siRNA conditions. Pre-treatment with the cytosolic Ca^2+^ buffer BAPTA-AM (50 µM) (n = 48 canaliculi) was used as a negative control (*p<0.05; n = 3 experiments). (**D**) and (**E**) Quantification of CMFDA canalicular accumulation under control (n = 163 canaliculi), scrambled siRNA (n = 73 canaliculi), BAPTA-AM (n = 111 canaliculi), scrambled siRNA+BAPTA-AM (n = 81 canaliculi), E-Syt1 siRNA+BAPTA-AM (n = 163 canaliculi), E-Syt2 siRNA+BAPTA-AM (n = 146 canaliculi) and E-Syt1+2 siRNA+BAPTA-AM (n = 164 canaliculi) conditions. (***p<0.0001; n = 3 experiments). Values are mean ± SD. Data were analyzed by one-way ANOVA, followed by Bonferroni's post-tests.

To investigate whether E-Syt knockdown affects hepatocyte secretory activity, we monitored apical organic anion secretion of the fluorescent Mrp2 substrate CMFDA by time-lapse confocal microscopy. Apical accumulation of CMFDA was quantified as an indicator of Mrp2 function (Figure 6a). This assay was used because it has been established previously that secretion of CMFDA in this system depends on expression of InsP3R-II and calcium signaling [11]. We observed that E-Syt1 and/or E-Syt2 siRNA-treated cells showed apical secretion kinetics that were similar to those of non-treated and scrambled siRNA-treated cells, whereas cells pre-treated with the cytosolic Ca^2+^ buffer BAPTA-AM had a significant decrease in apical CMFDA secretion (Figure 6b and c). Additionally, pre-treatment of E-Syt knockdown cells with BAPTA-AM did not change apical secretion kinetics compared to non-treated or scrambled siRNA-treated cells that were also treated with BAPTA-AM (Figure 6d and e). These data indicate that E-Syt1 and E-Syt2 do not participate in apical secretion of organic anions in hepatocytes.

### InsP3-induced Ca^2+^ signals are unaltered by knockdown of E-Syt

**Figure 7:**
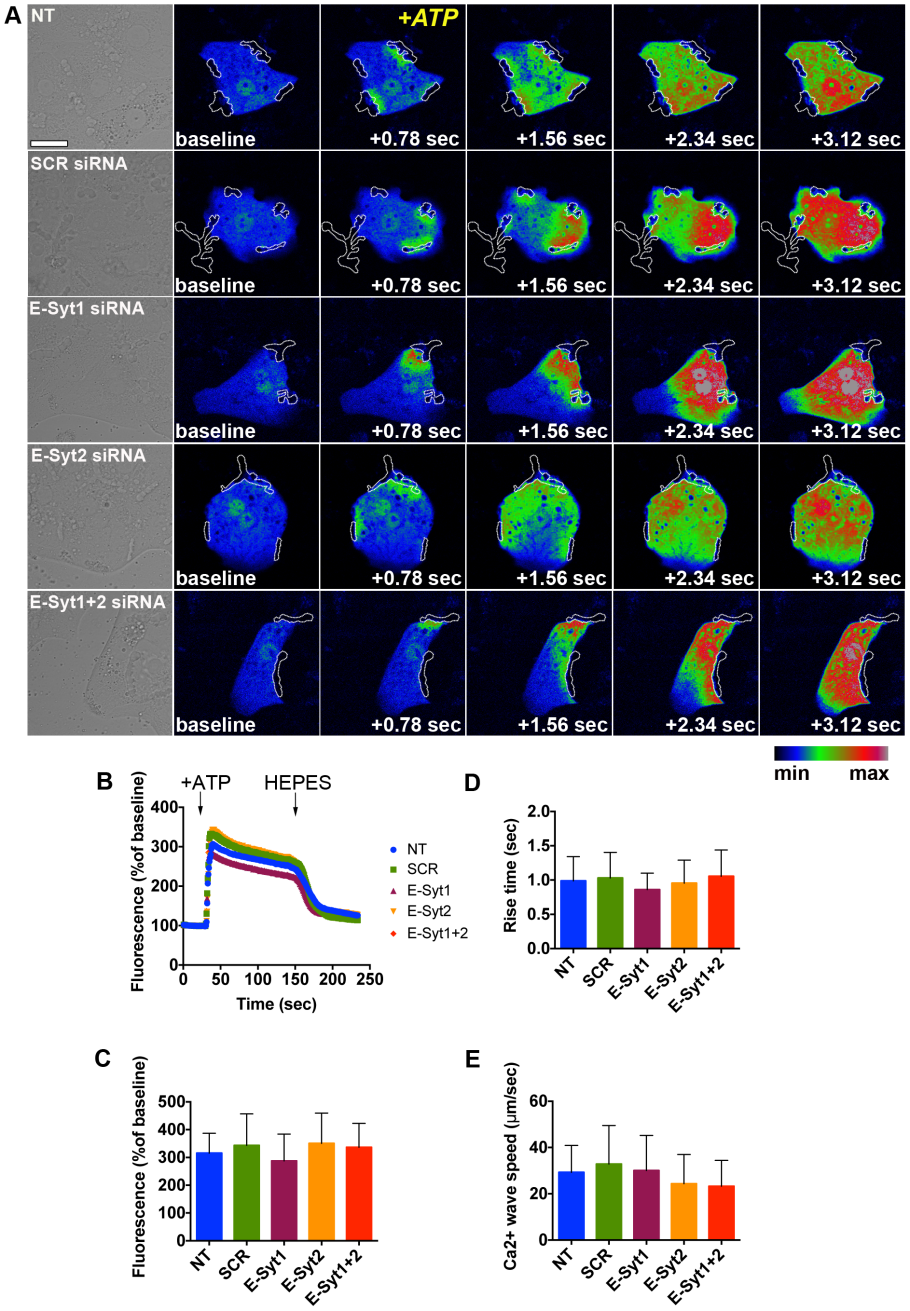
InsP3-induced Ca^2+^ signals are unaltered by E-Syt knockdown. (**A**) Representative DIC (left panels) and confocal fluorescence time-lapse images (right panels) of R-GECO-transfected, fully polarized rat hepatocytes under non-treated, scrambled, E-Syt1, E-Syt2 and E-Syt1+2 siRNA conditions and stimulated with 20µM ATP. Images were pseudocolored according to the scale shown at the bottom. Outlined structures represent the canalicular area of each cell, where Ca^2+^ waves begin. Scale bar = 20µm (**B**) Representative tracings from cells under each experimental condition are shown. Based on these tracings, peak signal amplitude (C), rise time (D) and Ca^2+^ wave speed (E) were measured. (n = 3 experiments, n = 20 cells per condition). Values are mean ± SD. Data were analyzed by one-way ANOVA, followed by Bonferroni's post-tests.

InsP3-induced Ca^2+^ signals begin as apical-to-basal Ca^2+^ waves in hepatocytes [27], and this pattern is disrupted when InsP3R-II redistributes away from the apical region [19]. To assess whether E-Syt knockdown alters the pattern of cytosolic Ca^2+^ signals in hepatocytes, we used the red fluorescent protein-tagged genetically encoded Ca^2+^ indicator for optical imaging R-GECO [30]. Fully polarized control, scrambled- and E-Syt siRNA-treated rat hepatocytes were stimulated with 20µM ATP and Ca^2+^ kinetics were analyzed by time- lapse confocal microscopy. ATP induced the formation of apical-to-basolateral Ca^2+^ waves (Figure 7a), similar to previous reports [4], [19], [32]. Signal amplitude, rise time and Ca^2+^ wave speed were unaltered in cells treated with E-Syt1 and/or E-Syt2 siRNA compared to non-treated or scrambled siRNA-treated cells (Figure 7b-e). Together, these results demonstrate that E-Syt1 and E-Syt2 do not play a role in InsP3-induced Ca^2+^ signaling in hepatocytes.

## Discussion

Apical clustering of InsP3Rs is important for the normal function of polarized epithelia, and loss of apical InsP3Rs appears to result in impaired secretion in both animal models of secretory disorders and in human disease. For example, loss of InsP3R-II in hepatocytes leads to impaired targeting of the apical membrane transporters Mrp2 [11] and bile salt export pump (Bsep) [10], and hence their secretory activity is compromised. Moreover, InsP3R-II expression is lost in both estrogen- and endotoxin-induced cholestasis [10], two different rodent models of human bile secretory disorders [35]. Furthermore, the expression of InsP3R-III, which is the main InsP3R isoform in bile duct epithelia, and which is also apically localized [6], is greatly reduced or absent in the bile ducts of patients with a range of cholestatic disorders including bile duct obstruction resulting from stone disease or malignancy, biliary atresia, primary biliary cirrhosis and sclerosing cholangitis [18]. Similarly, there is selective loss of InsP3R-III in bile ducts of animals subjected to bile duct ligation (BDL) or endotoxin injection, both accepted models of ductular cholestasis [18], [36]. Finally, InsP3R-II deficiency is associated with accumulation of zymogen granules and impaired secretion in pancreatic acinar cells [20]. Together, these observations provide evidence that InsP3R deficiency participates in the development of secretory disorders.

The importance of ER-PM contacts has been shown in such diverse processes as Ca^2+^ transport during excitation–contraction coupling in muscle cells [37], store-operated Ca^2+^ entry (SOCE) [38], [39], nonvesicular sterol lipid transport [40], [41], the regulation of phosphoinositide levels in the PM [42], [43], growth factor receptor signaling [44]–[46], vesicle trafficking and plasma membrane domain organization [47]. Therefore, this specialized inter-organelle communication is important to maintain proper lipid synthesis, protein folding, cell growth, polarity, hormone and calcium signaling, regulated secretion and endocytosis [47]. Inter-organelle signaling at ER–PM contacts may furthermore control cell–cell communication during normal cell development and disease states, such as tumor cell progression [47].

Our study shows that E-Syts do not participate in the tethering of InsP3R-II to the apical membrane in hepatocytes. However, finding other protein candidates that might be involved in this process is clinically relevant and remains an important question in order to better understand the pathogenesis of secretory diseases. Other possible ER-PM tethering candidates could be the vesicle-associated membrane protein-associated proteins (VAPs) or the TMEMs (or Anoctamins), which have been shown to establish ER-PM contacts in yeast [42], [48]. Whether these proteins play a role in InsP3R targeting to the apical membrane in secretory epithelia remains to be investigated.
